# Percutaneous tibial nerve stimulation versus sacral nerve stimulation for the treatment of faecal incontinence

**DOI:** 10.3389/fsurg.2024.1303119

**Published:** 2024-01-31

**Authors:** Alexander O’Connor, Elizabeth Reynolds, Clare Molyneux, Dipesh H. Vasant, Abhiram Sharma, Gemma Faulkner, John McLaughlin, Edward Kiff, Karen Telford

**Affiliations:** ^1^ Department of Colorectal Surgery, Manchester University NHS Foundation Trust, Manchester, United Kingdom; ^2^ Faculty of Biology, Medicine, and Health, The University of Manchester, Manchester, United Kingdom; ^3^ Neurogastroenterology Unit, Gastroenterology, Wythenshawe Hospital, Manchester University NHS Foundation Trust, Manchester, United Kingdom; ^4^ Division of Diabetes, Endocrinology and Gastroenterology, The University of Manchester, Manchester, United Kingdom; ^5^ Division of Diabetes, Endocrinology and Gastroenterology, The University of Manchester and Manchester Academic Health Sciences Centre, Manchester, United Kingdom

**Keywords:** faecal incontinence, multidisciplinary care, sacral neuromodulation, sacral nerve stimulation, percutaneous tibial nerve stimulation

## Abstract

**Introduction:**

Faecal incontinence (FI) is a common condition with a significant impact on quality of life (QoL). Neuromodulation treatments delivered by members of the multidisciplinary team including sacral nerve stimulation (SNS) and percutaneous tibial nerve stimulation (PTNS) are options for FI refractory to conservative management. The aim of this study was to assess whether a successful treatment with one neuromodulation modality corresponds with success in the other.

**Methods:**

A retrospective review of a prospectively managed neuromodulation database identified 15 patients who had undergone both PTNS and SNS. The definition of success of each treatment was a >50% improvement in any of The St. Mark's Incontinence Score, Manchester Health Questionnaire, or weekly faecal urgency or FI episodes.

**Results:**

Complete data from 12 patients was available for assessment and PTNS was delivered as the first treatment in nine patients. Overall, seven patients (58%) had successful PTNS treatment, with 10 (83%) having a successful SNS trials. Of the seven patients who had successful PTNS treatment, six patients (85.4%) went on to have success with SNS. Of the five patients who failed PTNS, four (80%) went on to have SNS success. Five (71%) of those who had positive PTNS outcomes had permanent SNS implantation as their final treatment decision.

**Conclusion:**

This study suggests that there is no clear relationship between successful PTNS treatment and an SNS trial period which may be explained by differing mechanisms of action or the potential placebo effect of PTNS. Further work is required to investigate any association in larger studies to inform clinical practice.

## Introduction

Faecal incontinence (FI) is a common condition afflicting 7% of all adults in the community. Its incidence increases with advancing age, female gender, and in the residential care population (1). FI carries a significant psychosocial and financial burden, along with profound negative effects on quality of life (QoL) whilst its successful treatment relies on an effective multidisciplinary team approach (2).

Neuromodulation is a treatment option for FI with percutaneous tibial nerve stimulation (PTNS) or sacral nerve stimulation (SNS) used where symptoms are refractory to conservative interventions (3–5). A permanent SNS implant is preceded by a two-week trial period of percutaneous nerve evaluation (PNE) with a temporary electrode connected to an external pulse generator (6). By contrast, PTNS is a minimally invasive, non-surgical treatment of initially up to 12 weekly 30 min sessions with percutaneous stimulation of the posterior tibial nerve (7).

The two forms of neuromodulation have never been compared in the same patients with FI to establish if an initial response in one corresponds with success in the other. This study aims to assess whether successful treatment with one modality can predict success with the other.

## Materials and methods

A retrospective review of a prospectively managed neuromodulation database at a tertiary pelvic floor unit based in Wythenshawe Hospital, Manchester, United Kingdom was performed to identify patients with FI who had undergone both PTNS and PNE. All patients had received maximum medical therapy and were discussed at the pelvic floor multidisciplinary team meeting before each treatment. Data was extracted including patient demographics, the dates and indications of neuromodulation treatment, and the FI outcome measures before and after each treatment. Patients were included if they were treated for the indication of faecal incontinence and were excluded if data was not available to determine the efficacy of either or both treatments.

PTNS therapy is delivered initially in 12 consecutive weekly 30-minute treatments using the Urgent PC® Neuromodulation system (Laborie®, NH, USA). Patients are placed in a seated position with their right leg elevated. The needle electrode is sited percutaneously 2 cm deep to the skin, 2 cm posterior to the tibia, and 5 cm cephalad to the medial malleolus. A surface electrode is placed on the ipsilateral limb to the medial aspect of the calcaneum. Correct placement is confirmed by eliciting either a motor response (plantar flexion of the great toe) or a sensory response (tingling to the toes, arch, or heel) through incremental increases in stimulation (7, 8).

In our unit, PNE is performed with a unipolar lead (Model 3057, Medtronic®, MN, USA) positioned at S3 under local anaesthetic using a standardised protocol. The trial period lasts two weeks for all patients. Following a successful trial, patients are offered permanent SNM implantation (Interstim® I (pre-2006)/Interstim® II (2006 onwards) System, Medtronic®, MN, USA).

The success of either treatment is defined as at least 50% improvement in any one of the FI specific St. Mark's Incontinence Score (SMIS), the FI specific QoL assessment the Manchester Health Questionnaire (MHQ), or weekly faecal urgency (FUE) or faecal incontinence episodes (FIE). The SMIS is a validated questionnaire capturing episodes of faecal leakage, incontinence, and urgency as well as accounting for the use of constipating medications or continence devices and the effect on daily activities (9). Scores range from ‘0’ representing perfect continence to “24” representing complete incontinence. The MHQ is a validated FI specific QoL questionnaire which measures QoL impact across nine domains: overall health, overall impact of FI on life, physical limitations, social limitations, relationship impact, emotional impact, sleep and energy impact, and overall FI severity (10). Scores range from “0” representing no adverse impact on an individuals’ QoL to a maximum score of “900”.

### Statistical analysis

Statistical analysis was performed using SPSS® for Mac® (version 29.0, IBM®, NY, USA). Data are presented as median (interquartile range) unless indicated. The Wilcoxon signed rank-sum test was used for paired comparisons of non-parametric data with statistical significance considered at *p* = <0.05 level.

## Results

**Figure 1:**
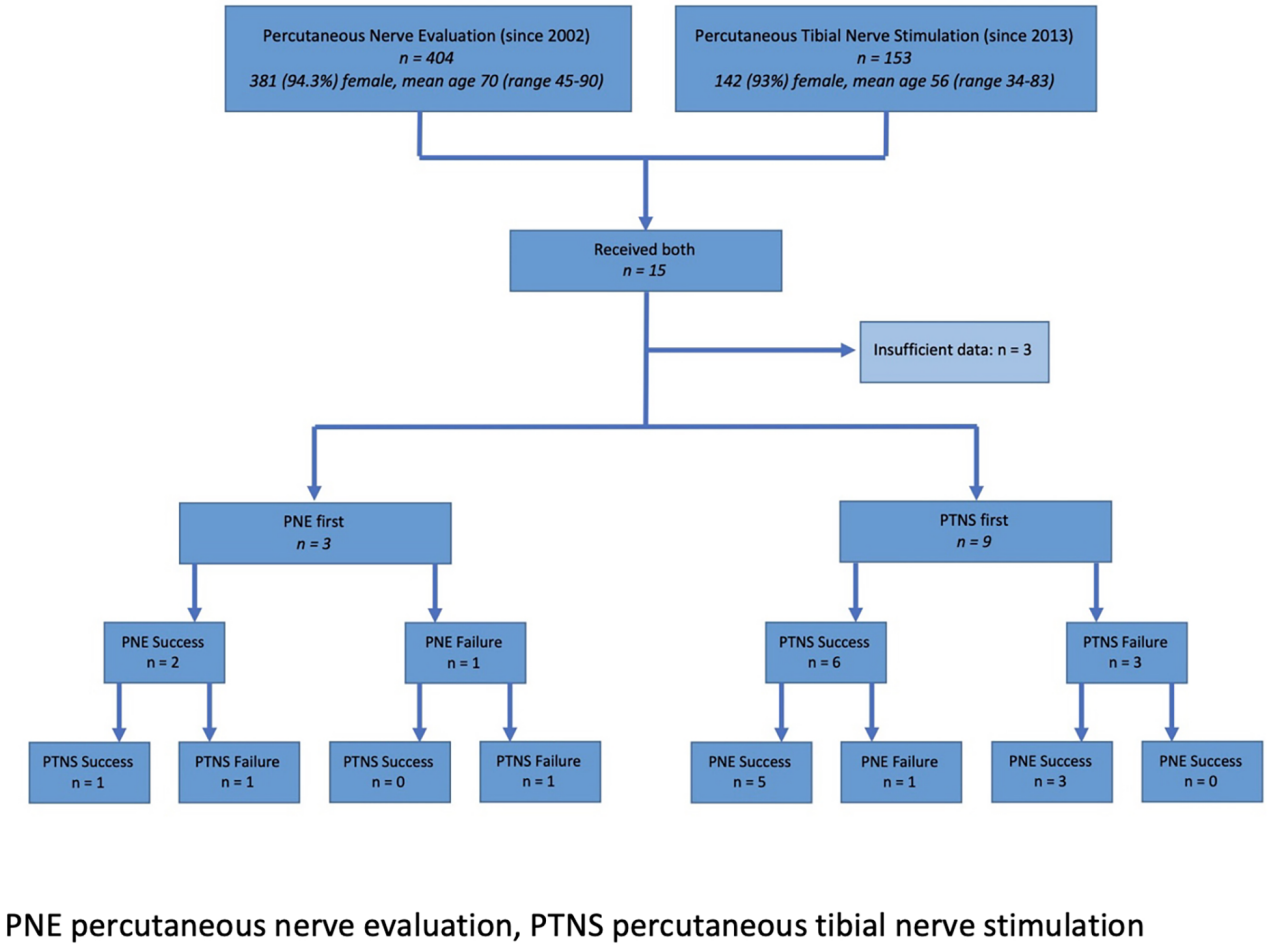
Included patient flow diagram.

In total, between May 2014 and November 2021, 12 patients were identified (Figure 1) with a mean age of 61 years [range 46–74, 12 (100%) female]. Patient demographics and symptoms are presented in Table 1. One patient only completed 11 of the 12 weekly sessions of PTNS with the remaining patients completing all 12 sessions. The median interval between treatments was 12 (8–36) months. Where PNE was performed first the subsequent course of PTNS was used in one patient where there was a delay in receiving a permanent SNS implant, and in two patients who wished to delay permanent implantation and undergo a less invasive treatment. Where PTNS was delivered as the first treatment, a subsequent PNE was performed due to ongoing symptoms that had not been adequately treated with PTNS.

**Table 1:** Included patient demographics.

Variable	Number of patients (*n* = 12)
Age, mean (range)	61 (46–74)
Gender, *n* (%)	Female: 12 (100%)
Obstetric history, *n* (%)	
Parous	11 (92%)
Vaginal delivery^a^	11 (100%)
Caesarean section^a^	1 (9%)
Episiotomies or perineal tear^b^	8 (73%)
Bowel function history, *n* (%)	
Faecal urgency	12 (100%)
Faecal urge incontinence	11 (92%)
Passive faecal incontinence	8 (67%)
Evacuatory difficulties	5 (42%)
Previous treatments for bowel dysfunction, *n* (%)	
Faecal incontinence surgery	0 (0%)
Transanal irrigation	1 (8%)
Endoanal ultrasound findings, *n (%)* [*n* = 11]	
Internal sphincter trauma^c^	4 (36%)
External sphincter trauma^c^	7 (64%)

^a^ Percentage calculated from parous females only.

^b^ Percentage calculated from females with vaginal deliveries only.

^c^ Percentage calculated from patients having undergone respective.

Baseline symptom severity measures for the included patients recorded before both PTNS and PNE are displayed in Table 2 and demonstrate a higher median FIE before PTNS (8.5 vs. 3.0, *p* = 0.003) but a lower median MHQ (564.17 vs. 636.60, *p* = 0.047) indicating a lower impact on QoL. There was no difference between baseline FUE and SMIS before each treatment.

**Table 2:** Baseline symptom severity measures recorded before percutaneous tibial nerve stimulation and peripheral nerve evaluation.

Outcome	PTNS *n* = 12	PNE *n* = 12	*p* ^a^
St mark's incontinence score	19 (16–20)	17 (14–19)	*p* = 0.35
Manchester health questionnaire	564.17 (379.17–648.33)	636.60 (565.80–688.30)	*p* = 0.047
Weekly faecal incontinence episodes	8.5 (4.5–11.75)	3 (1.25–5.25)	*p* = 0.003
Weekly faecal urgency episodes	17.5 (8.75–33.25)	10.5 (6.5–15.5)	*p* = 0.17

Results expressed as *median (interquartile range)*. Statistical significance considered at *p* < 0.05.

PNE, percutaneous nerve evaluation; PTNS, percutaneous tibial nerve stimulation.

^a^ Wilcoxon Signed Rank Test.

### Overall results

Overall, seven (7/12, 58%) patients reported successful PTNS treatment and ten (10/12, 83%) reported a successful PNE trial (Table 3, Supplementary Table 1). The overall success rate of all patients undergoing PTNS and PNE during the study period was 56% and 71% respectively.

**Table 3:** Overall treatment outcomes for individual patients.

Patient													
	1	2	3^a^	4	5	6	7	8	9	10^a^	11	12^a^	
PTNS result	Fail	Success	Fail	Fail	Success	Success	Success	Fail	Success	Fail	Success	Success	58% success (7/12)
PNE result	Success	Success	Fail	Success	Success	Success	Fail	Success	Success	Success	Success	Success	83% success (10/12)

PNE, percutaneous nerve evaluation; PTNS, percutaneous tibial nerve stimulation.

Success defined as >50% improvement in any of the St Mark's Incontinence Score, Manchester Health Questionnaire, weekly faecal urgency episodes or weekly faecal incontinence episodes.

^a^ Denotes PNE performed before PTNS in this patient.

Six patients (6/12, 50%) reported success with both PTNS and PNE, whilst only one patient (1/12, 8%) failed both therapies (Table 3).

Of the seven patients for whom PTNS was a success, six (6/7, 86%) also had a successful PNE trial. The one patient who failed subsequent PNE continued medical management at their own request. Of the ten patients who reported a successful PNE, six (6/10, 60%) also demonstrated a successful outcome with PTNS treatment. Six of those who reported PNE success (6/10, 60%) either received a permanent SNS implant, or were on the waiting list for one. Of the remaining four patients who had a successful PNE, one requested a colostomy whilst three chose to continue medical management.

### Percutaneous tibial nerve stimulation as first therapy

Nine patients received PTNS treatment first with six (6/9, 67%) reporting successful treatment. Five of these patients reporting success demonstrated at least a 50% reduction in FIE following PTNS with the remaining patient demonstrating a 43% reduction in FIE but a 71% reduction in FUE with PTNS (Supplementary Table 1). Despite achieving symptom improvement with PTNS they continued to demonstrate severe FI [median FIE: 4.0 (1.5–13)]. These patients therefore underwent subsequent PNE with a median treatment interval of 9 (7–13) months. There was however no significant difference between the outcome measures following PTNS and before PNE indicating that, any treatment benefit attributed to PTNS appeared to be maintained until their subsequent PNE (Table 4). The subsequent PNE was successful in eight (8/9, 89%) individuals overall and five (5/6, 83%) of those who reported initial PTNS success.

**Table 4:** Symptom severity measures recorded after percutaneous tibial nerve stimulation and before percutaneous nerve evaluation in the nine patients who received percutaneous tibial nerve stimulation as their first treatment.

Outcome	Post-PTNS *n* = 9	Pre-PNE *n* = 9	*p* ^a^
St mark's incontinence score	19 (13–21)	17 (15–20)	*p* = 0.888
Manchester health questionnaire	552.50 (515.63–646.87)	653.70 (572.67–694.53)	*p* = 0.310
Weekly faecal incontinence episodes	4.0 (1.5–13.0)	3.5 (1.5–5.0)	*p* = 0.515
Weekly faecal urgency episodes	20.0 (6.0–27.5)	13.0 (7.25–16.75)	*p* = 0.327

Results expressed as *median (interquartile range)*. Statistical significance considered at *p* < 0.05.

PNE, percutaneous nerve evaluation; PTNS, percutaneous tibial nerve stimulation.

^a^ Wilcoxon Signed Rank Test.

### Overall treatment efficacy

Of the seven patients who had a positive outcome following PTNS, four (4/7, 57%) had an improvement in two domains whereas three (3/7, 34%) had an improvement in only one domain. For those who had a successful PNE, five (5/10, 50%) had improvement in two domains and three (3/10, 30%) demonstrated improvement in three domains indicating that, in this cohort, PNE appeared to have the greatest overall beneficial impact on symptoms (Table 5).

**Table 5:** Distribution of improvement in outcome measures following treatment.

	PTNS *n* = 12	PNE *n* = 12
>50% improvement in weekly faecal urgency episodes	3	7
>50% improvement in weekly faecal incontinence episodes	6	9
>50% improvement in SMIS	1	3
>50% improvement in MHQ	1	2

MHQ, manchester Health Questionnaire; PNE, percutaneous tibial nerve stimulation; PTNS, percutaneous tibial nerve stimulation; SMIS, St. mark's incontinence score.

## Discussion

This study aimed to assess if successful treatment with one form of neuromodulation (PTNS or PNE) corresponded with success in the other for the treatment of FI in the same patient. It has demonstrated that successful treatment with PTNS may be associated with a successful PNE test, but that failure of PTNS treatment may not necessarily be associated with a failed PNE. This warrants further research in a larger study as it could be that PTNS success may be sufficient criteria for a permanent SNS implant, avoiding the need for a PNE test. However, a failure of PTNS treatment may not suggest that SNS will unsuccessful.

The mechanism of action of PTNS and its role in the management of FI is complex and poorly understood. It is considered a less invasive treatment option associated with reduced morbidity, but also reduced efficacy when compared to SNS despite displaying high patient satisfaction (8, 11). There are appreciable physiological effects with increased anal canal contractility and reduced rectal sensory thresholds having been observed (7). The recent randomised CONFIDeNT trial (CONtrol of Faecal Incontinence using Distal NeuromodulaTion) however could not identify a significant difference in patient symptoms when PTNS was compared to sham treatment (8). The results of this trial were later questioned as, when patients with concurrent obstructed defecation were excluded, a significant clinical effect of PTNS was observed compared to sham treatment (4). As a result, PTNS continues to be recommended for use in patients who fail conservative management (12). However, the potential for a “placebo” effect with PTNS received through weekly 30 min sessions with a trained continence therapist has not been completely excluded as the cause of any reported symptom improvements, including in this study (13). This may explain our findings that failure to achieve success with PTNS did not necessarily mean failure of PNE. Most patients (9/12, 75%) in our study underwent PTNS as their first neuromodulation therapy earlier in their treatment pathway at our tertiary unit which would explain why this group started PTNS treatment with a higher median FIE than before their subsequent PNE trial (8.5 vs. 3.0, *p* = 0.003). These patients will have continued to receive maximum conservative management guided by a team of specialist therapists during their PTNS treatment, the effect of which has not yet been quantified in clinical trials. Indeed, this may explain why the reported benefit following PTNS persists until undergoing PNE after a median of 9 (7–13) months (Table 4) despite cessation of PTNS therapy.

SNS is currently recommended as the first-line surgical treatment for FI after failure of conservative management (14) with expanding indications to include patients with anal sphincter trauma and low anterior resection syndrome (15, 16). Despite this, the mechanism of action is not fully understood; however, it has been demonstrated to modulate afferent neural activity to the pelvic floor along with efferent activity to the central nervous system (3). It is unclear if PTNS and SNS share the same mechanism of action given the selective stimulation of leg nerves during PTNS which is actively avoided in SNS therapy (6). If both therapies do however share the same mechanism, the neurostimulation with SNS is delivered immediately adjacent to the target nerve root which conceivably delivers a stronger direct stimulation and may explain the greater clinical effect following PNE reported in this study. Without a defined mechanism of action, patient selection is reliant on the outcome of PNE. If a potential association between PTNS and PNE success is confirmed in clinical trials, previous PTNS success could be considered sufficient criteria for permanent SNS implantation. Alternatively, PTNS could be offered as an effective treatment option before SNS (14), or to those patients waiting for SNS surgery which have suffered delays in recent years (17).

There is sparse literature available investigating PTNS and SNS together for FI. In one study of 20 patients who failed ongoing PTNS therapy for FI the authors reported that subsequent PNE was successful in 14 (70%) individuals. They suggested this may be a result of the more central and direct action of SNS resulting in more intensive stimulation assuming both therapies share the same mechanism of action (18). A similar study investigating patients treated with both modalities for overactive bladder identified that PTNS failure did not predict subsequent SNS failure, however only one patient had a successful PTNS treatment in their cohort making generalisable conclusions difficult (19). A further small prospective cohort study of 19 patients compared the efficacy of SNS (*n* = 10) or PTNS (*n* = 9) in male FI patients and described both treatments demonstrated similar rates of success (SNS: 9/10, PTNS: 7/9). It was however considered that this finding could be due to the different aetiology of FI in men compared to women (20).

Our study demonstrates that, consistent with other reports, neuromodulation can offer significant benefits to patients with FI. Although the mechanism of action of either PTNS or SNS is not fully understood and may be different, these techniques in combination warrant further exploration to establish their location in the treatment pathway for FI. Of the seven patients who reported successful PTNS treatment, six (6/7, 86%) received, or are awaiting, a permanent SNS implant in our study. If these results were replicated in a larger clinical trial, it may suggest that a successful PTNS treatment could correlate with a positive outcome from subsequent SNS treatment. In our study we identified six patients who received PTNS treatment earlier in their treatment pathway and demonstrated at least a 50% improvement in symptoms with PTNS treatment. However, they continued to suffer with severe FI requiring SNS treatment which was successful in five (5/6, 83%) patients. It may therefore be considered that PTNS could be used both as a therapeutic option and a diagnostic tool to highlight patients who may find subsequent SNS treatment efficacious if their FI symptoms remain severe.

This study is the first reported series of patients with FI treated successfully with both PTNS and SNS. However, it has several limitations, principally the small number of patients at a tertiary unit and its retrospective design making the generalisability of the findings questionable.

## Conclusion

Successful treatment with PTNS may be associated with a successful PNE and referral for permanent implantation, however no clear association between the two modalities has been identified, as 80% (4/5) of patients who failed PTNS went on to have a successful PNE. This apparent contradiction may suggest that PNE delivers a more direct and stronger stimulation, or that there is indeed a “placebo” rather than clinical effect from PTNS treatment. Further work is required to establish the mechanism of action of PTNS and SNS and establish their role in the treatment of FI. If an association between these modalities exist with a shared mechanism of action, PTNS could be used to both treat patients and highlight those for whom SNS might be a transformative management option for FI if symptoms remain severe.

## Data Availability

The original contributions presented in the study are included in the article/Supplementary Material, further inquiries can be directed to the corresponding author.
