# Supplementary material for: Percutaneous tibial nerve stimulation versus sacral nerve stimulation for the treatment of faecal incontinence
Source: Front Surg. 2024 Jan 31;11:1303119. doi: 10.3389/fsurg.2024.1303119 (PMC10864541; doi:10.3389/fsurg.2024.1303119)
Supplement: Supplementary file 1 [file Table1.docx]

Supplementary Table 1: A detailed summary of patient outcomes from both percutaneous tibial nerve stimulation treatment and peripheral nerve evaluation.

| Patient | 1 | | 2 | | 3* | | 4 | | 5 | | 6 | |
| --- | --- | --- | --- | --- | --- | --- | --- | --- | --- | --- | --- | --- |
| St Mark’s Incontinence Score | Pre PTNS | Post PTNS | Pre PTNS | Post PTNS | Pre PTNS | Post PTNS | Pre PTNS | Post PTNS | Pre PTNS | Post PTNS | Pre PTNS | Post PTNS |
|  | 21 | 21 | 20 | 14 | 14 | 17 | 10 | 11 | 20 | 16 | 21 | 20 |
|  | Improvement = 0% | | Improvement = 30% | | Improvement = -21% | | Improvement = -10% | | Improvement = 20% | | Improvement = 5% | |
|  | Pre PNE | Post PNE | Pre PNE | Post PNE | Pre PNE | Post PNE | Pre PNE | Post PNE | Pre PNE | Post PNE | Pre PNE | Post PNE |
|  | 15 | 12 | 13 | 8 | 14 | 10 | 15 | 15 | 18 | 9 | 22 | 9 |
|  | Improvement = 20% | | Improvement = 38% | | Improvement = 29% | | Improvement = 0% | | Improvement = 50% | | Improvement = 59% | |
| Manchester Health Questionnaire | Pre PTNS | Post PTNS | Pre PTNS | Post PTNS | Pre PTNS | Post PTNS | Pre PTNS | Post PTNS | Pre PTNS | Post PTNS | Pre PTNS | Post PTNS |
|  | 430 | 530 | 691.67 | 237.50 | 514.17 | 610 | 379.17 | 670.83 | 329.17 | N/A | 687.5 | 575 |
|  | Improvement = -23% | | Improvement = 66% | | Improvement = -19% | | Improvement = -77% | | Improvement = N/A | | Improvement = 16% | |
|  | Pre PNE | Post PNE | Pre PNE | Post PNE | Pre PNE | Post PNE | Pre PNE | Post PNE | Pre PNE | Post PNE | Pre PNE | Post PNE |
|  | 565.80 | 532.50 | 727.4 | 725 | 654.17 | 487.5 | 636.6 | 670.83 | 593.3 | 464.9 | 670.8 | 485.8 |
|  | Improvement = 6% | | Improvement = 0.3% | | Improvement = 25% | | Improvement = -5% | | Improvement = 22% | | Improvement = 28% | |
| Weekly Faecal Incontinence Episodes | Pre PTNS | Post PTNS | Pre PTNS | Post PTNS | Pre PTNS | Post PTNS | Pre PTNS | Post PTNS | Pre PTNS | Post PTNS | Pre PTNS | Post PTNS |
|  | 7 | 11 | 2 | 0 | 4 | 6 | 6 | 15 | 11 | 1 | 14 | 8 |
|  | Improvement = -57% | | Improvement = 100% | | Improvement = -50% | | Improvement = -150% | | Improvement = 91% | | Improvement = 43% | |
|  | Pre PNE | Post PNE | Pre PNE | Post PNE | Pre PNE | Post PNE | Pre PNE | Post PNE | Pre PNE | Post PNE | Pre PNE | Post PNE |
|  | 2.5 | 0.5 | 2 | 0 | 0 | 0 | 3.5 | 1 | 1 | 0 | 5.5 | 0 |
|  | Improvement = 80% | | Improvement = 100% | | Improvement = 0% | | Improvement = 71% | | Improvement = 100% | | Improvement = 100% | |
| Weekly Faecal Urgency Episodes | Pre PTNS | Post PTNS | Pre PTNS | Post PTNS | Pre PTNS | Post PTNS | Pre PTNS | Post PTNS | Pre PTNS | Post PTNS | Pre PTNS | Post PTNS |
|  | 35 | 32 | 4 | 6 | 63 | 82 | 11 | 13 | 28 | 20 | 17 | 5 |
|  | Improvement = 9% | | Improvement = -50% | | Improvement = -30% | | Improvement = -18% | | Improvement = 29% | | Improvement = 71% | |
|  | Pre PNE | Post PNE | Pre PNE | Post PNE | Pre PNE | Post PNE | Pre PNE | Post PNE | Pre PNE | Post PNE | Pre PNE | Post PNE |
|  | 15.5 | 14 | 13 | 0.5 | 0 | 0 | 6.5 | 3 | 41.5 | 3 | 15.5 | 2 |
|  | Improvement = 10% | | Improvement = 96% | | Improvement = 0% | | Improvement = 54% | | Improvement = 93% | | Improvement = 87% | |
|  | | | | | | | | | | | | |
| Patient | 7 | | 8 | | 9 | | 10* | | 11 | | 12* | |
| St Mark’s Incontinence Score | Pre PTNS | Post PTNS | Pre PTNS | Post PTNS | Pre PTNS | Post PTNS | Pre PTNS | Post PTNS | Pre PTNS | Post PTNS | Pre PTNS | Post PTNS |
|  | 18 | 19 | 20 | 12 | 15 | 21 | 18 | 18 | 18 | 19 | 19 | 9 |
|  | Improvement = -6% | | Improvement = 40% | | Improvement = -40% | | Improvement = 0% | | Improvement = -6% | | Improvement = 53% | |
|  | Pre PNE | Post PNE | Pre PNE | Post PNE | Pre PNE | Post PNE | Pre PNE | Post PNE | Pre PNE | Post PNE | Pre PNE | Post PNE |
|  | 17 | 17 | 21 | 5 | 14 | 16 | 17 | 14 | 19 | 14 | 19 | 13 |
|  | Improvement = 0% | | Improvement = 76% | | Improvement = -14% | | Improvement = 18% | | Improvement = 26% | | Improvement = 32% | |
| Manchester Health Questionnaire | Pre PTNS | Post PTNS | Pre PTNS | Post PTNS | Pre PTNS | Post PTNS | Pre PTNS | Post PTNS | Pre PTNS | Post PTNS | Pre PTNS | Post PTNS |
|  | 648.33 | 535.83 | 564.17 | 569.17 | 611.67 | 755 | N/A | 358.8 | 610 | 510.84 | 246.67 | 225.83 |
|  | Improvement = 17% | | Improvement = -1% | | Improvement = -23% | | Improvement = N/A | | Improvement = 16% | | Improvement = 8% | |
|  | Pre PNE | Post PNE | Pre PNE | Post PNE | Pre PNE | Post PNE | Pre PNE | Post PNE | Pre PNE | Post PNE | Pre PNE | Post PNE |
|  | 468.3 | 440.8 | 688.3 | 293.3 | 696.6 | 611.6 | 371.67 | 332.5 | N/A | N/A | 575.8 | 181.6 |
|  | Improvement = 6% | | Improvement = 57% | | Improvement = 12% | | Improvement = 11% | | Improvement = N/A | | Improvement = 68% | |
| Weekly Faecal Incontinence Episodes | Pre PTNS | Post PTNS | Pre PTNS | Post PTNS | Pre PTNS | Post PTNS | Pre PTNS | Post PTNS | Pre PTNS | Post PTNS | Pre PTNS | Post PTNS |
|  | 31 | 4 | 12 | 18 | 6 | 3 | 3 | 3 | 11 | 2 | 10 | 2 |
|  | Improvement = 87% | | Improvement = -50% | | Improvement = 50% | | Improvement = 0% | | Improvement = 82% | | Improvement = 80% | |
|  | Pre PNE | Post PNE | Pre PNE | Post PNE | Pre PNE | Post PNE | Pre PNE | Post PNE | Pre PNE | Post PNE | Pre PNE | Post PNE |
|  | 4.5 | 8.5 | 0.5 | 0.5 | 4 | 0 | 2.5 | 0.5 | 7.5 | 2.5 | 6.5 | 2 |
|  | Improvement = -89% | | Improvement = 0% | | Improvement = 100% | | Improvement = 80% | | Improvement = 67% | | Improvement = 69% | |
| Weekly Faecal Urgency Episodes | Pre PTNS | Post PTNS | Pre PTNS | Post PTNS | Pre PTNS | Post PTNS | Pre PTNS | Post PTNS | Pre PTNS | Post PTNS | Pre PTNS | Post PTNS |
|  | 47 | 23 | 13 | 25 | 18 | 30 | 8 | 8 | 23 | 6 | 5 | 3 |
|  | Improvement = 51% | | Improvement = -92% | | Improvement = -67% | | Improvement = 0% | | Improvement = 74% | | Improvement = 40% | |
|  | Pre PNE | Post PNE | Pre PNE | Post PNE | Pre PNE | Post PNE | Pre PNE | Post PNE | Pre PNE | Post PNE | Pre PNE | Post PNE |
|  | 8 | 8.5 | 4 | 0.5 | 18 | 6.5 | 9.5 | 2.5 | 11.5 | 9 | 6.5 | 1 |
|  | Improvement = -6% | | Improvement = 88% | | Improvement = 64% | | Improvement = 74% | | Improvement = 22% | | Improvement = 85% | |

PTNS percutaneous tibial nerve stimulation, PNE percutaneous nerve evaluation

** Denotes PNE performed before PTNS in this patient.*
